# Supplementary material for: Causal Relationship between Aging and Anorexia Nervosa: A White-Matter-Microstructure-Mediated Mendelian Randomization Analysis
Source: Biomedicines. 2024 Aug 16;12(8):1874. doi: 10.3390/biomedicines12081874 (PMC11351342; doi:10.3390/biomedicines12081874)
Supplement: Supplementary file 1 [file biomedicines-12-01874-s001.zip › Supplementary Figure/Figure S8/Leaveoneout plot of MR analysis for TL to ubm-b-2027.pdf]

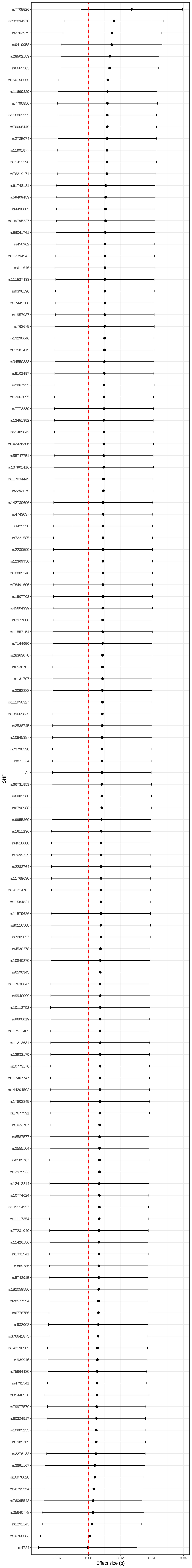
rs7705526
rs202034370
rs2763979
rs9419958
rs28502153
rs6669563
rs150150565
rs11699829
rs7790856
rs116863223
rs76666449
rs3785074
rs11991877
rs11412296
rs76219171
rs61748181
rs59409453
rs4498805
rs139795227
rs56061761
rs450962
rs112394943
rs611646
rs111527438
rs9398196
rs17445108
rs1957937
rs762679
rs13230646
rs73581419
rs34550383
rs8102497
rs2967355
rs13062095
rs7772289
rs12451892
rs61405042
rs142426306
rs55747751
rs137901416
rs117034449
rs2293579
rs142730696
rs4743037
rs429358
rs7221585
rs2230590
rs12369950
rs10805346
rs78491606
rs1907702
rs45604339
rs2977608
rs11557154
rs7164950
rs28363070
rs6536702
rs131797
rs3093888
rs111950327
rs139669835
rs2538745
rs10845387
rs73730598
rs871134
A8
rs66731853
rs6881568
rs6790988
rs9955360
rs1611236
rs4616688
rs7099229
rs2282764
rs11769630
rs141214782
rs11584821
rs11579626
rs80116508
rs7209057
rs4530278
rs10840270
rs6590343
rs117630647
rs9940099
rs10112752
rs9600019
rs117512405
rs11212631
rs12932178
rs10773176
rs117407747
rs144204502
rs17803849
rs17677991
rs1023767
rs6587577
rs2555104
rs8105767
rs12925933
rs12412214
rs10774624
rs145114957
rs11117354
rs77231040
rs114266156
rs1332941
rs869785
rs5742915
rs182059586
rs28577594
rs6776756
rs932002
rs376641875
rs143190905
rs939916
rs75664430
rs4731541
rs35446936
rs79977579
rs80324517
rs10905255
rs1985369
rs2276182
rs3891167
rs16978028
rs56799554
rs76065543
rs35640778
rs1291143
rs10768683
rs4724
SNP
−0.02
0.00
0.02
0.04
0.06
Effect size (b)
